# Computational Investigations on Soundproof Applications of Foam-Formed Cellulose Materials

**DOI:** 10.3390/polym11071223

**Published:** 2019-07-23

**Authors:** Carmen Debeleac, Petronela Nechita, Silviu Nastac

**Affiliations:** 1 Research Center for Mechanics of Machines and Technological Equipments, Engineering and Agronomy Faculty, “Dunarea de Jos” University of Galati, 810017 Braila, Romania; 2 Department of Environmental, Applied Engineering and Agriculture, Engineering and Agronomy Faculty, “Dunarea de Jos” University of Galati, 810017 Braila, Romania

**Keywords:** foam-formed materials, cellulose, soundproof, poroacoustics

## Abstract

Recent studies have highlighted an innovative way to produce highly porous materials based on cellulose fibers. These studies have focused on the foam-forming process, where the cellulose fibers and other components are mixed with foam. In the authors’ previous research, the foam-formed cellulose materials (FCM) were obtained by mixing a surfactant with cellulose fibers, taken from virgin pulp and recovered papers. In the present paper, the authors performed additional experimental and computational analyses in order to evaluate the sound insulation capabilities of these FCM beyond the initial impedance of tube investigations. The poroacoustics computational methodology parameters—i.e., airflow resistivity, porosity, tortuosity, viscous, and thermal characteristic lengths—were herein evaluated. This analysis was performed using both a theoretical/empirical approach from the specialized literature and an experimental investigation developed by the authors. The computational investigations were conducted in two stages: First, we evaluated the approximation of the experimentally gained normal incidence parameters, in terms of absorption and reflection, respectively, relative to the estimated ones. The second stage of analysis consists of a parametrical estimation of sound insulation characteristics concerning the incidence angle of sound hitting the porous layer. The results presented in this paper are in agreement with the computational experimental results, providing extended soundproof characteristics to the incidence angle of the acoustic field. Further, this study supplies additional information useful for future analyses regarding the influences of random geometry air inclusions into the FCM layer.

## 1. Introduction

Sound absorbing materials with low density (e.g., polystyrene foam) are often used for protection and insulation applications. However, bio-based alternatives for low-density insulation materials are needed to replace the fossil-based ones [1]. The development of biomaterials based on highly recyclable and biodegradable lignocellulosic fibers have attracted great interest in the composite material science community as a viable alternative to petroleum-based materials [2,3]. Besides their biological process control on waste management, these materials have many important applications in the fields of sound and energy absorption, thermal insulation, radiation shielding, filtration, packaging, building industry, electronics, and medicine. The performances of lignocellulosic materials in sound or thermal insulation applications can be improved by their porous structure, as porous materials are generally used as sound absorbing materials due to their efficiency in attenuating acoustic energy. Generally, porous materials have a high sound absorption coefficient compared to other materials [4].

Due to a lack of manufacturing processes, cellulose fibers are rarely used as raw material for obtaining porous or low-density materials in insulating applications. Yet recent research has highlighted an innovative way to produce highly porous materials based on cellulose fibers. This method includes a foam-forming process where cellulose fibers and other components are mixed with a foaming agent instead of water, as is common in conventional methods. The result is a lightweight material with low-density that is mechanically pressed with a load that increases strength [5,6].

When comparing with fiber products obtained using a water forming process (e.g., conventional papermaking process), the high vacuum levels and wet pressing are not used and drying is done by non-restrained conditions or non-contact methods to prevent the fiber network from collapsing [7].

Moreover, the use of foam as a carrier in manufacturing fibrous materials has recently been intensively studied. It was first developed in the 1960s to reduce the amount of pulp fibers needed to compare traditional wet-forming paper products [8,9]. In the 1970s, experiments were conducted with foam instead of water as the medium in which fibers were suspended [10,11].

Additionally, this innovative technology is supported by potential savings in terms of energy and water, especially when compared with conventional water forming fibrous materials [12]. The other important advantage is the three-dimensional structure of foam that prevents the flocculation of fibers and allows production of high porosity products with unique properties [13].

The effect of various parameters such as air content, thickness, porosity, and consistency of the foam-formed composites was reported in many studies [14]. Increasing the thickness and consistency of the product resulted in an increase of acoustic dampening in the foam-formed material.

Independent of their composition, many insulating materials are either porous and/or fibrous. In thist study, the acoustic absorption characteristics of these kinds of materials were investigated on a large scale. Many previous papers have focused on underlining factors that influence absorption performance of natural fibrous and sound absorbing materials. Physical properties such as internal porosity, density, and thickness establish relationships between their geometrical characteristics. This includes open porosity, fiber radius, and fiber orientation, and how each can contribute to the sound absorption performance of natural fibers [15,16,17,18,19,20]. In addition, characteristics such as flow resistivity, absorption coefficient, propagation coefficient, characteristic impedance, wavenumber for several porous, and/or fibrous materials are determined using experimental tests or numerical simulations [21,22,23]. In the case of porous materials, sound absorption properties are dependent upon frequency, composition, thickness, surface finish, and the method of mounting [24]. In addition, mechanical characterizations of the poroelastic materials (i.e., polyurethane foam, recycled rubber, polyurethane resin, etc.) have been comprehensivly analyzed [25,26,27,28]. The reference papers show the importance of structural vibration in polyurethane foam, as the characteristic impedance and propagation of constant absorption vary the mechanical parameters like density, Young’s modulus, Poisson’s coefficient, and the structural damping factor, all characteristics that have influence on resonance frequency absorption.

On the other hand, there has been a lot of research interested in developing eco-friendly materials with high sound absorption performances, that could potentially solve environmental problems (i.e., noise and environmental pollution). Zhu et al. [29] studied the recent advancements in sound transmission properties of bio-based materials and multilayered structures based on a co-extruded wood plastic composite using sound absorption and insulations products. Egab et al. [30] presented the physical parameters that provide a link between acoustical and material properties, as well as current experimental methods used to measure these parameters. For soundproof applications, the use of recyclable cellulose loose-fill composite materials represents an alternative solution to conventional methods for obtaining the fibrous composites. Many researchers have showed how acoustic measurements have excellent sound insulation performance on materials such as recycled cellulose fibers (i.e., luffa, yarn waste, coconut, and Kenaf fiber) [31,32,33,34,35,36]. The sound absorption coefficients of flax fiber fabric and its reinforced composite (i.e., balsa wood) were predicted by Zhang and Shen [37] using a double-porosity model, which showed a superior sound absorption performance (especially at high frequency) compared to the synthetic materials-based sandwich structure composite that contributed to their multi-scale structures of sound wave attenuation and energy dissipation. Mamtaz et al. [38] indicated the importance of the pretreatment of virgin natural fibers and especially how moisture contents, thicker diameter, and lower antifungal quality helped obtain higher acoustic absorption properties of these materials.

Further, empirical or multi-parameters models (the Delany–Bazley model, Biot model, Miki model, and Johnson–Champoux–Allard models, etc.) are utilized on a large scale for the prediction of the sound absorption coefficient considering the porosity and flow resistivity of the tested materials [39,40,41]. On the other hand, the inverse approach of material identification based on acoustical performances (absorption coefficient or flow resistance) has been tested and simulated using experimental data via the Bayesian approach [37,42,43].

Remarkable acoustical approaches on a large area of materials was performed using the COMSOL software, wherein we investigated the acoustic properties (i.e., velocity and temperature) of fibrous media at different scales [20,44,45]. Semeniuk and Göransson [46] modelled the interaction of the coupled viscous and thermal boundary layers on a representative array of cylindrical lightweight fibrous porous materials. Brennan et al. [47] studied the influence of permeability on wave propagation in an open-cell polyurethane foam-based Kelvin foam model with regular-sized cell-holes. In addition, this software helped investigate Multiphysic modeling of sound absorption in porous media based on their geometry [20,48,49].

Using the the Representative Volume Element (RVE) approach, numerical methods like the Finite Element Method (FEM) and the Boundary Element Method (BEM) provide powerful tools to predict the acoustic behavior of sound absorbing materials. However, these methods are known to be extremely computationally demanding, especially when the frequency increases or when the dimensions of the domain are too large and are thus limited to low frequency applications. For example, the relationship between a mechanical response and an internal microstructural morphology of porous [17] and fibrous media [20] can be investigated with RVE theory. The airflow in impacted polyurethane foam was successfully investigated by Mills and Lyn [50], as well as Mills [51], across a foam block. Further, they also used FEA to model impact compression. Moreover, porous materials with a single- and multi-layer predicted the acoustic behavior combining both FEM and BEM methods [42]. Hyperelastic behavior of polymer soft foams was put into evidence by using the FEM simulation [52].

In our study, foam-formed cellulose materials (FCM) were obtained by mixing a surfactant with cellulose fibers and then tested with promising results on sound absorption applications [36]. Other than the basic acoustical investigations, we performed an additional experimental and computational analyses in order to evaluate the sound insulation capabilities of these bio-based materials. The parameters used in the poroacoustics computational methodology, such as airflow resistivity, porosity, tortuosity, viscous, and thermal characteristic lengths have been evaluated. This step of analysis was performed based on both the theoretical and empirical approach presented in the specialized literature, as well as on the experimental investigations developed by the authors.

## 2. Materials and Methods 

### 2.1. Background Approaches

The basic computational model configuration was considered to be a rectangular geometry air domain model, in which an incident sound field hits the porous FCM layer, backed by a sound hard wall, at a normal incidence angle. The incident field was simulated by applying a background pressure field to the air domain. The model contains only a small portion and the extension of the domain to infinity was approached by applying periodic Floquet boundary conditions on both side boundaries. Opposite of the FCM layer, a perfectly matched layer (PML) domain was used to simulate an infinitely larger air domain. The thickness of the porous layers was used within the initial acoustical tests based on impedance tube method, with respect to the correspondent values of the samples. The poroacoustics models used within the simulations were the Delany–Bazley (D-B) and Johnson–Champoux–Allard (JCA) models. The last model was considered with its basic formulation and extended version, namely the Johnson–Champoux–Allard–Pride–Lafarge (JCAPL). Within this analysis, the FCM layer was supposed have uniform distribution, with homogenous poroacoustics characteristics in the entire domain. We constructed a working hypothesis because an analytical solution was available. Thus, we conducted a multiple comparison between experimental, computational, and analytical results.

The aim of the model is to facilitate a comparison between the experimental and computational soundproofing properties of FCM, both in terms of normal sound incidence and frequency. More specifically, it was intended to study the specific surface impedance and the absorption and the reflection coefficients. 

The explicit configuration of the model was considered because it enabled the simulations of the porous material with some various shape air inclusions. The validation of the explicit configuration model was conducted using an additional set of models in which the FCM layer was simply modeled as an impedance boundary of the porous layer type and backed by a sound-hard wall. This approach was only used to validate the effects of a normally incidence acoustic field on the porous layer, which is also compared to in the experimental results.

The second direction of analysis considered how the background acoustical pressure field was supposed to hit the porous layer at different angle values (specifically 30, 45, and 60 degrees). A parametric simulation was performed, in terms of the incident angle, for each case of material characteristics and layer configuration.

### 2.2. Materials

The computational analyses were performed on three types of foam-formed cellulose materials (see Table 1), with 15–17 mm thickness and 36–38 kg/m^3^ density. These FCM were obtained from bleached hardwood cellulose (BHCF), with 100–200 µm average fiber length, and 15–35 µm diameters from recycled fibers from recovered papers (RCF), dry grinded to obtain a fluffy material with high content of fines.

Sodium dodecyl sulphate (SDS) and C_12_ H_25_SO_4_Na are commonly used in cosmetics products and was added as anionic surfactant into the liquid before mixing.

### 2.3. Methods

#### 2.3.1. Foam Forming Methods

**Figure 1:**
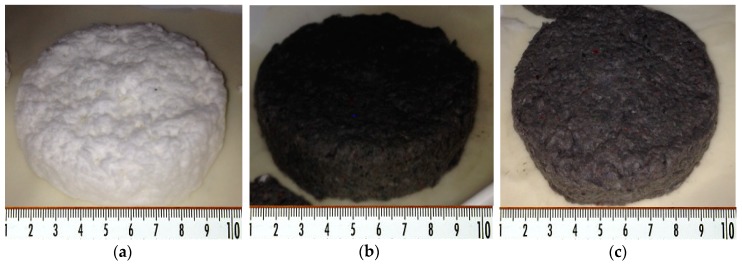
Snapshots of wet structures for the foam-formed cellulose materials within this study: (**a**) sample P1; (**b**) sample P3S; and (**c**) sample P4. [36].

Foam can be made in many ways [53,54,55]. In our study, foam was generated using an axially agitated mix. The cellulose fibers (i.e., virgin cellulose fibers and recycled fibers) with a pre-determined weight to obtain a slurry pulp with 2.0% consistency, were soaked overnight (24 h) in distilled water with 1% sodium hydroxide (1N concentration). Then, the pulp slurry was mixed at high shear velocity (2000 rpm) for 15 min. Before mixing, 2% wt (to the pulp weight) surfactant SDS was added. The dispersion of foam was mixed until 60% air content was obtained. The air content from pulp foam dispersion was periodically calculated during the agitation process by comparing the mass of a known volume of foamed slurry pulp (*m_foam_*) to the mass of the fiber/water suspension. *M_pulp_* was required to obtain the same volume (1 − (*m_foam_*/*m_pulp_*)) [56]. The foam and fibers suspension were filtered and dewatered using a Buchner funnel (15 cm diameter) with a filter paper at the bottom. The filtering was developed at a low level and vacuumed for approximately 20 min. Dewatered FCM with wet structure is presented in Figure 1 [36].

#### 2.3.2. Microscopic Studies and Structural Analysis

**Figure 2:**
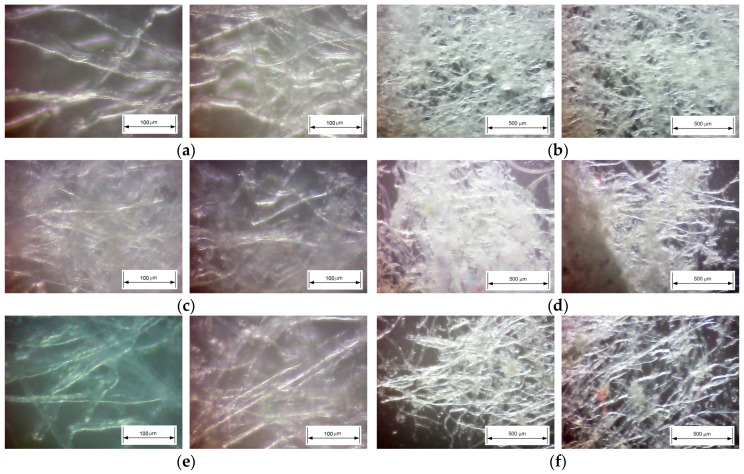
Optical micrographs of foam/water formed cellulose materials: sample P1 with magnification 350x (**a**) and 80x (**b**); sample P3S with magnification 350x (**c**) and 80x (**d**); sample P4 with magnification 350x (**e**) and 80x (**f**).

The micrographs of foam-formed cellulose materials (dried at 23 °C and 56% relative humidity) were obtained using an optical microscope (Science ETD Microscope, Bresser GmbH, Rhede, Germany) with 80× respectively 350x magnification and CMOS camera. Selected pictures are presented in Figure 2.

The thickness and density of foam formed cellulose materials were performed based on standardized methods specific to paper products (ISO 534: Paper and Board–Determination of thickness and apparent density).

#### 2.3.3. Acoustics Evaluation

The sound absorption properties were evaluated in terms of reflection coefficient *R*(*ω*):

(1) $$R\left(\omega \right)=\frac{Z_{s}\left(\omega \right)-Z_{0}}{Z_{s}\left(\omega \right)+Z_{0}},$$

and the absorption coefficient *α*(*ω*):

(2) $$\alpha \left(\omega \right)=1-{\left|R\left(\omega \right)\right|}^{2},$$

with the surface acoustic impedance *Z_s_*(*ω*) given by:

(3) $$Z_{s}\left(\omega \right)=-i\;Z_{c}\left(\omega \right)\;\cot\left(k_{c}\left(\omega \right)\;d\right),$$

where the characteristic impedance *Z_c_*(*ω*) is [43]:

(4) $$Z_{c}\left(\omega \right)=\frac{1}{\varepsilon_{p}}\sqrt{\rho \left(\omega \right)\;K\left(\omega \right)},$$

The complex wave number *k_c_*(*ω*) is [43]:

(5) $$k_{c}\left(\omega \right)=\omega \sqrt{\frac{\rho \left(\omega \right)}{K\left(\omega \right)}},$$

where *ρ*(*ω*) denotes dynamic fluid density, *K*(*ω*) the dynamic bulk modulus, *ε_p_* the porosity, *ω* the angular frequency, *Z*_0_ = (*ρ*_0_ *c*_0_) the impedance of the air saturating the pores, (*ρ*_0_) the density of air, *c*_0_ the speed of sound in air, and *d* the sample thickness.

Computational investigations regarding the evaluation of sound proofing properties for the proposed FCM was performed using the poroacoustics theoretical approach based on the available empirical models. Hence, we were able to describe the sound absorbing characteristics (wave impedance and the characteristic sound propagation constant, respectively) in terms of materials’ physical properties, such as porosity, tortuosity, and airflow resistivity. Within this study, we considered both microstructural and phenomenological models. Microstructural schematization was developed according to classical D-B model, which is adequate for highly porous materials and have enough accuracy for a large range of materials, but provides unrealistic predictions for very high or too low frequencies [30]. 

On the other hand, the phenomenological models have the ability to simulate porous materials that account for their random geometry. From the group of phenomenological models, the JCA model was adopted, which correlates with the effective density and bulk modulus, respectively, of the porous layer, to the five physical parameters. In addition, the JCAPL model was considered, which takes into account the other three physical parameters compared in the JCA model.

The microstructural D-B model was the first empirical model proposed in 1970 by Delany and Bazley. Its general formulation [30,57] supposed the following expression of the characteristic impedance:

(6) $$Z_{c}=Z_{0}\left(1+C_{1}\;X^{-C_{2}}-i\;C_{3}\;X^{-C_{4}}\right),$$

The complex wave number is given by:

(7) $$k=\frac{\omega }{c_{0}}\left(1+C_{5}\;X^{-C_{6}}-i\;C_{7}\;X^{-C_{8}}\right),$$

with the parameter *X* defined as:

(8) $$X=\frac{\rho_{0}f}{\sigma },$$

where *f* is the frequency, *ω* is the angular frequency, *i*^2^ = −1, and *σ* denotes air flow resistivity. The eight constants (*C*_1..8_) acquire various values according to different materials. For different types of materials, the values presented in Table 2 can provide accurate predictions. Note that the first-row values correspond to the basic D-B model.

A major inconvenient of the D-B model is that the applicability is restricted by the porosity value close to one (0.01 < X < 1) and (1000 < σ < 5000) [30].

The phenomenological JCA porous matrix model is defined by the complex dynamic rigid density, *ρ*(*ω*):

(9) $$\rho \left(\omega \right)=\frac{\tau_{\infty }\rho_{f}}{\varepsilon_{p}}\left(1+\frac{\sigma \varepsilon_{p}}{i\omega \rho_{f}\tau_{\infty }}\sqrt{1+\frac{4i\omega \tau_{\infty }^{2}\mu \rho_{f}}{\sigma_{}^{2}\Lambda^{2}\varepsilon_{p}^{2}}}\right),$$

and the equivalent bulk modulus *K*(*ω*):

(10) $$K\left(\omega \right)=\frac{\gamma \;p_{A}}{\varepsilon_{p}}{\left[\gamma -\left(\gamma -1\right)\;{\left(1+\frac{8\mu }{i\omega \;\Lambda^{\prime^{2}}P_{r}\rho_{f}}\sqrt{1+\frac{i\omega \;\Lambda^{\prime^{2}}P_{r}\rho_{f}}{16\mu }}\right)}^{-1}\right]}^{-1},$$

where *τ*_∞_ is the tortuosity factor, *ρ_f_* is the fluid density, *ε_p_* is the porosity, *σ* is the airflow resistivity, *μ* is the dynamic viscosity, *p_A_* is the quiescent pressure, *γ* is the ratio of specific heats, Λ is the viscous characteristic length, Λ’ is the thermal characteristic length, and *P_r_* denotes the Prandtl number.

The JCAPL model introduces proper corrections to the dynamic density and bulk modulus, respectively, at low frequencies, through viscous and thermal behavior, which are not captured by the JCA model. The JCAPL model has the complex dynamic rigid density, given by:

(11) $$\rho \left(\omega \right)=\frac{\rho_{f}\tilde{\tau }\left(\omega \right)}{\varepsilon_{p}},$$

and the complex bulk modulus:

(12) $$K\left(\omega \right)=\frac{\gamma \;p_{A}}{\varepsilon_{p}}\frac{1}{\tilde{\beta }\left(\omega \right)},$$

With:

(13) $$\tilde{\tau }\left(\omega \right)=\tau_{\infty }\left(1+\frac{1}{i\bar{\omega }}\tilde{F}\left(\omega \right)\right),\;\tilde{F}\left(\omega \right)=1-P+P\;\sqrt{1+\frac{M}{2\;P^{2}}i\bar{\omega }},$$

(14) iω¯=iωρfk0τ∞μεp, M=8 k0τ∞εpΛ2, P=M4 (τ0τ∞−1)=2 k0τ∞2εpΛ2(τ0−τ∞),

(15) $$\tilde{\beta }\left(\omega \right)=\gamma -\left(\gamma -1\right)\;{\left(1+\frac{1}{i\bar{\omega^{\prime }}}\tilde{F^{\prime }}\left(\omega \right)\right)}^{-1}=\gamma -\left(\gamma -1\right)\;\tilde{\tau^{\prime }}{\left(\omega \right)}^{-1},$$

(16) $$\tilde{F^{\prime }}\left(\omega \right)=1-P^{\prime }+P^{\prime }\;\sqrt{1+\frac{M^{\prime }}{2\;P^{\prime^{2}}}i\bar{\omega^{\prime }}},\;i\bar{\omega^{\prime }}=\frac{i\omega \rho_{f}P_{r}{k^{\prime }}_{0}}{\mu \varepsilon_{p}},\;M^{\prime }=\frac{8\;{k^{\prime }}_{0}}{\varepsilon_{p}\Lambda^{\prime^{2}}},\;P^{\prime }=\frac{M^{\prime }}{4\;\left({\tau^{\prime }}_{0}-1\right)},$$

where the new parameters are static viscous tortuosity (also coined inertial factor), *τ*_0_, and static thermal tortuosity *τ*’_0_ (both dimensionless), respectively. Static viscous permeability is defined as *k*_0_ = *μ*/*σ* and static thermal permeability, *k*’_0_ (both have SI unit m^2^). Generally, *τ*_0_ ≥ *τ*_∞_; for porous materials it can be supposed that *τ*_0_ = 3/4 *τ*_∞_ and *τ*’_0_ ≈ *τ*_0_/*τ*_∞_. Regarding static thermal permeability, *k*’_0_ ≥ *k*_0_; it was assumed that for the cylindrical pores, *k*’_0_ = *k*_0_, in the same time that for slits *k*’_0_ = *τ*_∞_ *k*_0_. It has to be mentioned that the JCA model is recovered by setting *M*’ = *P* = *P*’ = 1.

#### 2.3.4. Geometrical Properties

In order for a material to be soundproofing efficient, it has to provide a structure to transfer the energy into and an acceptable range of porosity so that the sound waves can penetrate far enough into the respective structure to allow for multiple interactions within it. Open porosity (*ε_p_*) is one of the most important parameters, along with material density and speed of sound, in tailoring the performance of an acoustic absorber [23].

(17) $$\varepsilon_{p}=\frac{V_{pore}}{V_{total}}=1-\frac{\rho_{a}}{\rho_{m}},$$

where *V_pore_* and *V_total_* denotes the pore and total volumes respectively (m^3^), *ρ_a_* is the density of porous material (kg/m^3^), and *ρ_m_* is the solid part density (skeletal density of the solid matrix) of the material (kg/m^3^). 

In order to describe the viscous forces and the thermal exchanges between the porous frame and its saturating fluid at high frequencies, the phenomenological models introduced two parameters that account for the thermal and viscous losses, which appear at the acoustic boundary layer at pore walls, namely the viscous and thermal characteristic lengths.

The viscous characteristic length, Λ, is related to the size of the interconnection between the two pores in the porous material and controls the viscous effects at medium and high frequencies. Its expression is given by:

(18) $$\Lambda =\frac{1}{s}\sqrt{\frac{8\mu \tau_{\infty }}{\varepsilon_{p}\sigma }},$$

wherein *s* denotes the pore geometry dependent factor, with typical values between 0.3 and 3.0 (literature provides *s* = 1 for circular, *s* = 1.07 for square, *s* = 1.14 for triangular pores, respectively, and *s* = 0.78 for slits) [58].

The second characteristic dimension, the thermal characteristic length Λ’, is related to the size of the pores in the porous material and controls the thermal effects at medium and high frequencies It is defined as a doubled ratio between the volume (*V_pore_*) and the surface area (*S_pore_*) of the pores

(19) $$\Lambda^{\prime }=\frac{2V_{pore}}{S_{pore}}.$$

Generally, Λ ≤ Λ’, cylindrical pores present a special case where Λ = Λ’, and a good approximation supposes 2Λ = Λ’ and *s* = 1, which can be used in order to derive simpler formulations for the sound propagation in rigid-framed fibrous materials.

**Figure 3:**
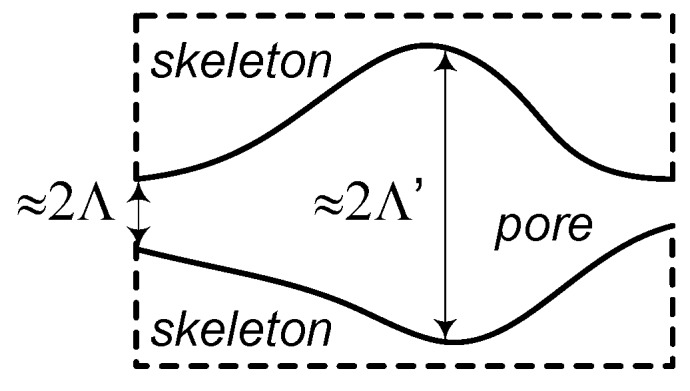
Schematization used for physical description of viscous and thermal characteristic lengths.

Figure 3 provides a simple schematization of the physical signification of the viscous and thermal characteristic lengths, respectively.

If it is assumed that fibers do not overlap, then Λ’ can be evaluated in respect to the characteristic fiber radius *r_f_* and open porosity, respectively [16]:

(20) $$\Lambda^{\prime }=r_{f}\frac{\varepsilon_{p}}{1-\varepsilon_{p}}.$$

If the fibers overlap, from the Equation (20) results an additional term in denominator of Equation (21), which are difficult to evaluate (it depends, among other things, on the number of fibers intersections and their corresponding shapes). However, the influence of the intersections can be considered by a correction term, *ξ*, which represents the fiber intersection intensity, within the denominator of Equation (21) as follows [16]:

(21) $$\Lambda^{\prime }=r_{f}\frac{\varepsilon_{p}}{1-\varepsilon_{p}+\xi }.$$

#### 2.3.5. Transport Properties

The tortuosity is an important non-acoustical property that determines the high-frequency acoustical behavior of a porous layer. Physically speaking, the tortuosity accounts for the twistiness of pores in a material and is a measure of fiber deviation from normal course, relating to the ratio of the actual path (*h_e_*) traveled by a high frequency sound through the porous sample to the sample thickness *d*:

(22) $$\tau_{\infty }≅\frac{h_{e}}{d}.$$

The simplest way to estimate the tortuosity in the material porosity is as follows:

(23) $$\tau_{\infty }=\frac{1}{\sqrt{\varepsilon_{p}}}.$$

Allard, in 1994, assumed that the thickness of the viscous boundary layer was still considerably smaller than the characteristic pore dimension for high frequencies. Thus, the phase velocity of the sound wave within the porous medium approaches:

(24) $$c_{b}≅\frac{c_{0}}{\sqrt{\tau_{\infty }}}.$$

An experimental measurement of tortuosity can be performed through evaluation of the phase speed of an ultrasound wave within the sample by comparing the time delay between the wave fronts of the incident and the transmitted waves. Considering Allard’s assumptions (and that for typical sound barriers materials with average pore sizes around 100 μm) these will be attained for frequencies higher than 25 kHz. The authors have adopted this method to evaluate the FCM tortuosity. It has to be noted that this method has wide utilization and is related to its relative facilely implementation in practice. 

**Figure 4:**
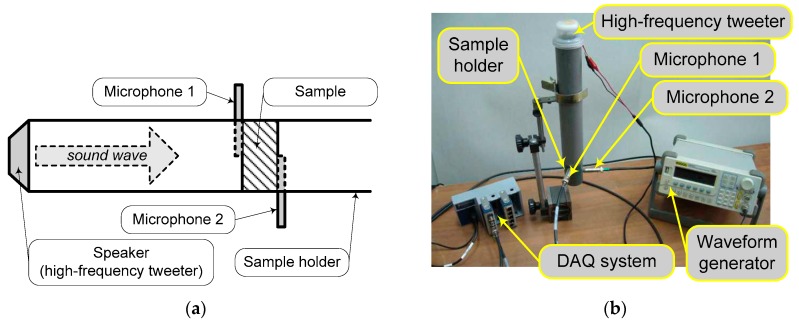
Experimental laboratory setup for tortuosity evaluation: (**a**) Schematic diagram of the experimental setup; (**b**) general view of the laboratory setup with ultrasonic generator and sample holder. Component parts are marked on pictures, where DAQ denotes the Data Acquisition system (National Instruments, Austin, Texas, USA).

A schematic diagram of the method (a), in addition with an image of the laboratory setup (b), is depicted in Figure 4. It was used an ultrasonic tweeter, able to provide a high range of frequency, up to 40 kHz, mounted on a side of a sample holder tube. The sample holder enables the measurement of the incident and transmitted wave near the both sides of the material sample. The setup has to provide aligned centers of sound transducers, as well as sample and ultrasonic wave generators. Considering the speed of sound *c*_0_, the microphones diameter *d_m_*, the sample thickness *d*, and evaluated the time delay Δ*t*, the tortuosity results by the expression:

(25) $$\tau_{\infty }={\left(\frac{c_{0}\;\Delta t-d_{m}}{d}\right)}^{2},$$

During the experimental tests, the authors used acoustical transducers with *d_m_* = 6.35 × 10^−3^ m, an exciting signal with 35 kHz frequency. The average values for samples tortuosity are shown in Table 3 for P1, P3S, and P4 materials. Considering the literature provided, values of 95% and 96% accuracy for the experimental method enable a good correlation to the values gained by the empirical expression (see Equation (23)).

Even if the flow resistance is able to characterize the acoustic performances for regular application with a homogeneous fiber and open-cell foam materials, the flow resistivity and the porosity bring additional information and can completely specify the acoustical properties. This is due to them being used within the empirical models intended for evaluation of impedance and wave number, respectively [23].

Thus, the airflow resistivity becomes an important parameter in soundproofing properties in the proposed FCM. It has to be mentioned that fibrous materials widely present anisotropy. However, in this study, the authors considered only the flow resistivity across the normal direction to the sample and the experimental tests were performed according to the direct airflow method, provided within ISO 9053-1:2018 (acoustics, determination of airflow resistance, Part 1: Static airflow method).

**Figure 5:**
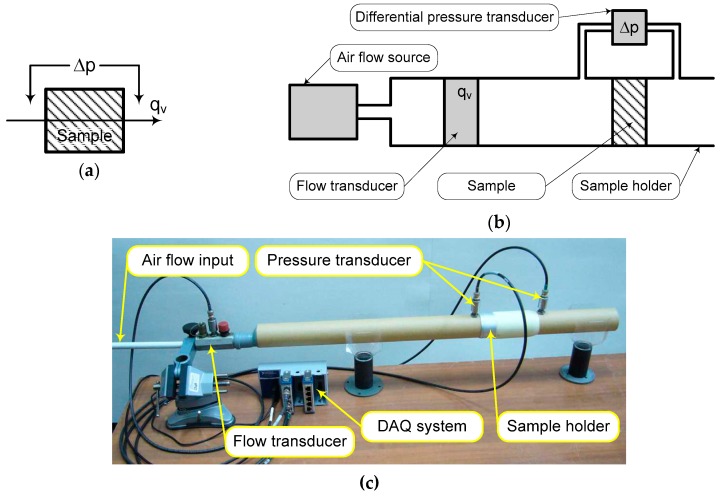
Experimental laboratory setup for airflow resistivity evaluation: (**a**) Basic principle; (**b**) schematic diagram of the experimental setup underlining the main parts; and (**c**) general view of laboratory setup with component parts marked on picture.

According to the schematics in Figure 5, which present the basic principle (a) and the configuration of laboratory experimental setup (b) for airflow resistance measurement, it is easy to evaluate the airflow resistance *R_f_* of a material sample [23]:

(26) $$R_{f}=\frac{\Delta p}{q_{v}},$$

where Δ*p* (Pa) denotes the static pressure drop across the material sample and *q_v_* (m^3^/s) denotes the volumetric flow rate through material sample. Taking into account the specific flow resistance, *R_s_* is as follows:

(27) $$R_{s}=R_{f}A,$$

with *A* (m^2^) denotes the cross-sectional area of the sample (normal to the flow direction) and results in air flow resistivity:

(28) $$\sigma =\frac{R_{s}}{d},$$

where *d* (m) is the thickness of material sample.

Regarding the theoretical estimation of the airflow resistivity, the literature provides various empirical laws. Based on the material type applicability, the authors adopted two expressions, which are presented as follows.

The airflow resistivity *σ*, with the material apparent density *ρ_m_* and the mean fiber diameter *d_f_*, could be estimated by the following empirical expression [34]:

(29) $$\sigma =\frac{K_{2}\;\rho_{m}^{K_{1}}}{d_{f}^{2}},$$

where constants *K*_1_ and *K*_2_ depends on material type. For *K*_1_, the literature proposes 1.404 (related to the Garai and Pompoli model, polyester fibers) and 1.530 (related to the Bies and Hansen model), respectivley. For glass-wool-like composites, the literature recommended that *K*_2_ be valued at 3.18 × 10^−9^.

Carman and Kozen proposed a model derived from the Poiseuille’s equation for laminar flow, which enabled the link between the static airflow resistivity, the porosity, and the mean fiber diameter [59]:

(30) $$\sigma =\frac{180\mu \;{\left(1-\varepsilon_{p}\right)}^{2}}{d_{f}^{2}\;\varepsilon_{p}^{3}},$$

where μ is the dynamic viscosity and μ = 1.81 × 10^−5^ (Pa s).

Supposing the circular section of fibers with a mean radius is valued at 13 × 10^−6^ m and the constant *K*_1_ tuned to 1.59, the airflow resistivity for each FCM sample is presented in Table 3. In addition, the relative errors of the experiment and of the average value were evaluated. In order to perform the computational analysis, as well as taking into account the comparative values of relative errors in Table 4, the authors adopted the value of airflow resistivity for each sample as a rounded mean between the experimental and, respectively, the theoretical estimation according to Equations (29) and (30).

## 3. Results

Comparing and summarizing the assertions presented in previous sections of this paper, the authors proposed a set of specific characteristics for the FCM in order to perform the computational analyses. These characteristics take into account the requirements of the empirical models. Its values are presented in Table 5. 

The computational investigations were conducted in two stages. First, we evaluated the approximation of the experimentally gained normal incidence parameters (in terms of absorption and reflection respectively) to the estimated ones. After tuning the process of the simulation model, it was continued with a second stage of analysis, which consisted of parametrically estimating sound absorption and reflection parameters, respectively, with respect to the incidence angle of sound hitting the porous layer. The results will be separately presented within the following subsections.

### 3.1. Comparison Between Experiments and Computational Approaches

The sound insulation characteristics, in terms of absorption and reflection coefficients, were experimentally evaluated based on two set of tests, using the method of impedance tube with four microphones. Each set of tests corresponded with large and small diameter tubes. These results were presented in detail [36] and had specifications within ISO 10534-2:1998 (acoustics, determination of sound absorption coefficient and impedance in impedance tubes, Part 2: Transfer-function method). In addition, the computational estimations were conducted with respect to the empirical models: D-B, JCA, and JCAPL (briefly presented in Section 2.3.3). 

**Figure 6:**
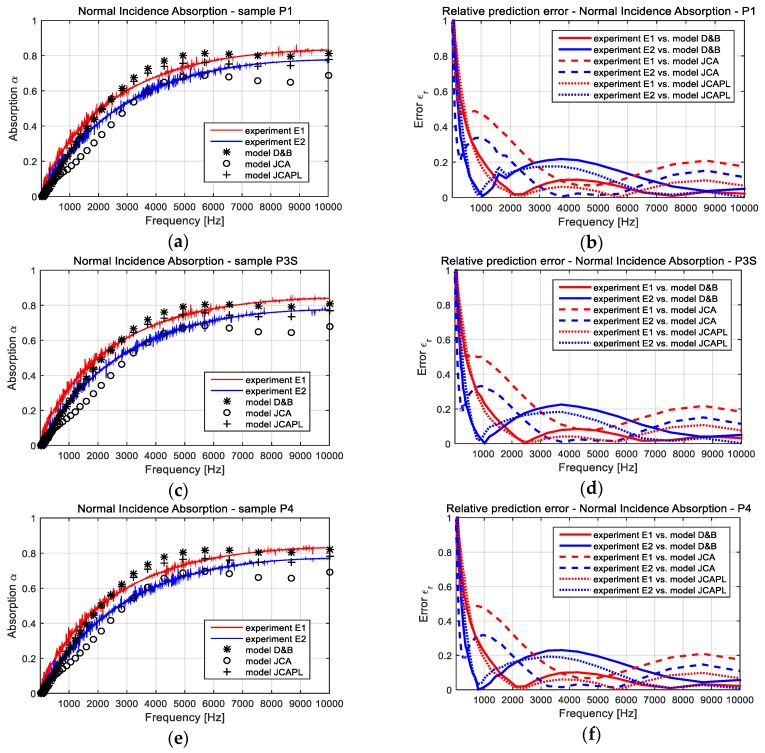
Comparative results between experimental evaluated and, respectively, computational estimated normal incidence absorption coefficients: (**a**) Absorption for sample P1; (**b**) Relative prediction error for sample P1; (**c**) Absorption for sample P3S; (**d**) Relative prediction error for sample P3S; (**e**) Absorption for sample P4; (**f**) Relative prediction error for sample P4. The significations of the symbols within graphs were mentioned on each diagram legend.

**Figure 7:**
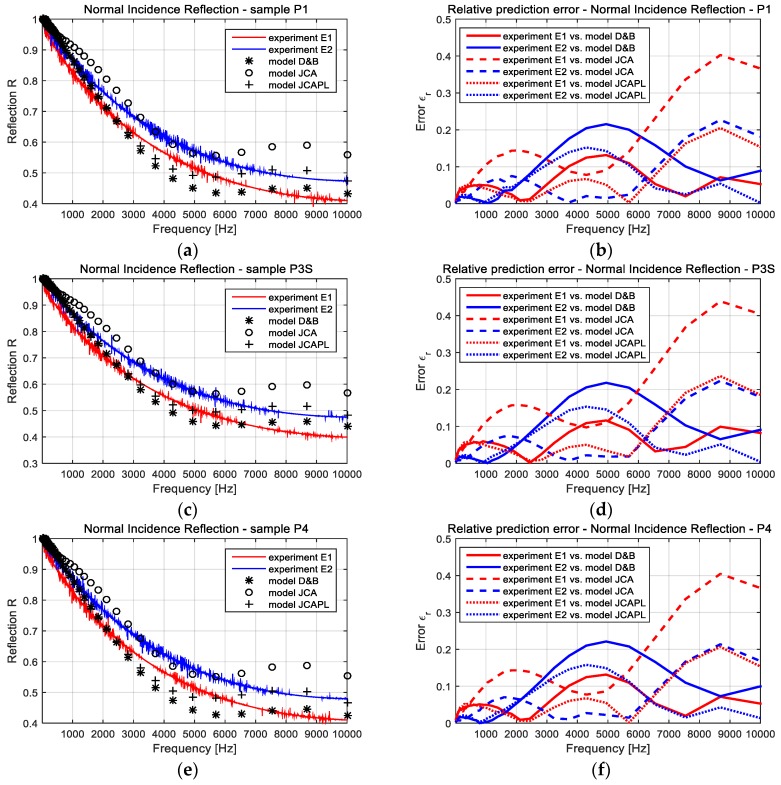
Comparative results between evaluated and computational estimated normal incidence reflection coefficients. (**a**) Reflection for sample P1; (**b**) relative prediction error for sample P1; (**c**) reflection for sample P3S; (**d**) relative prediction error for sample P3S; (**e**) reflection for sample P4; and (**f**) relative prediction error for sample P4. The significations of the symbols within graphs were mentioned on each diagram legend.

The ensemble of all these results were presented in Figure 6—regarding the absorption, and, respectively, in Figure 7—regarding the reflection characteristics. For each monitored parameter (α and *R*), it was additionally evaluated the relative prediction error of the experiment to the theory estimation. The diagrams within Figure 6 and Figure 7 depict a comparative evolution of the insulation characteristics, in respect to the frequency, and separately for each sample (namely P1, P3S and P4). The meanings of each diagram and graph were mentioned on title and legend respectively.

### 3.2. Extended Computational Investigations

The second stage of investigations within this study consisted of an extension of computational analyses with respect to the incidence angle of the sound wave at the interface between the air domain and the porous FCM layer. Actually, this additional analysis highlights the advantage of a proper computational model, harmonized to the realistic behavior in minimizing the experimental tests volume and reducing the investigation’s length of time.

**Figure 8:**
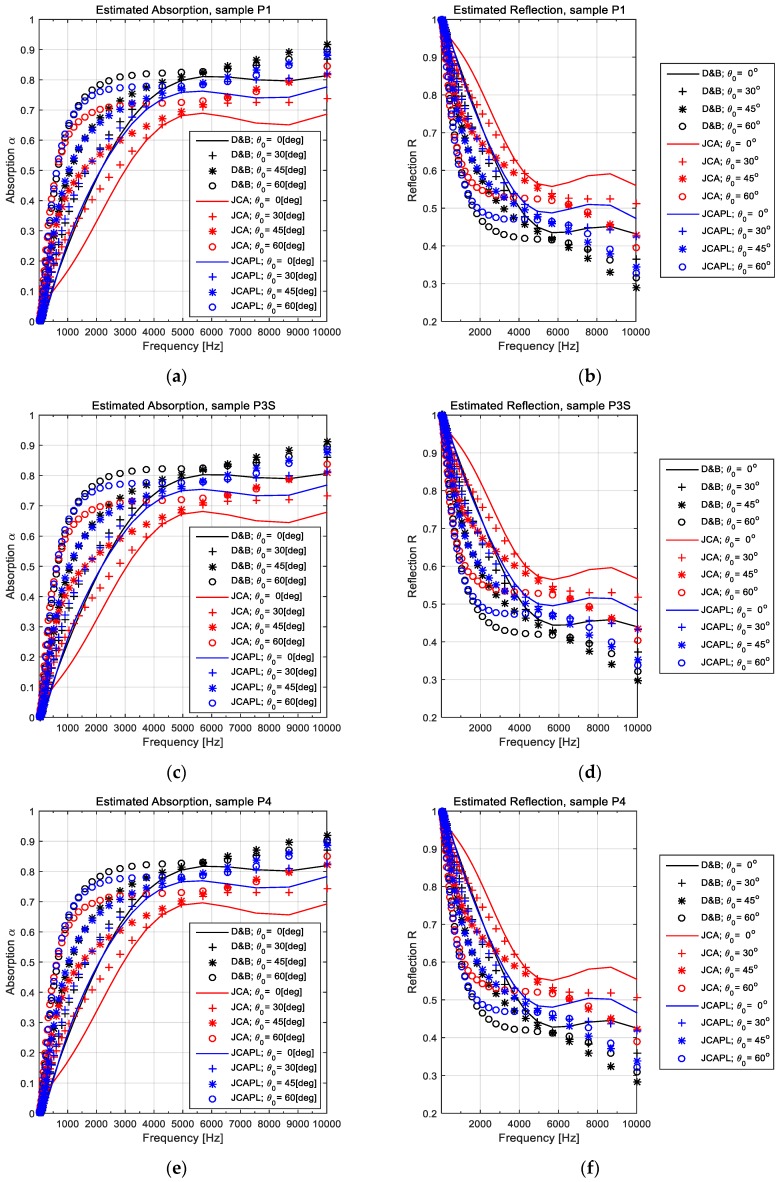
Parametrical evolutions of absorption α and reflection *R* coefficients respectively, with respect to the sound incidence angle θ_0_. (**a**) α for sample P1; (**b**) *R* for sample P1; (**c**) α for sample P3S; (**d**) *R* for sample P3S; (**e**) α for sample P4; and (**f**) *R* for sample P4. The significations of the symbols within graphs were mentioned on each diagram legend.

The results were depicted in Figure 8, as comparative diagrams with respect both to the FCM sample and the estimated soundproof parameter (α or *R*). The values of the incidence angle θ_0_ were discretely adopted within the range of 0–60 degrees. For the sake of comparison, the graphs within Figure 8 were plotted with continuous line for normal incidence and with graphical markers for the other value of θ_0_ (30°, 45°, 60°). The meanings of each diagram and symbol were mentioned on title and legend, respectively.

## 4. Discussion

Analyzing the micrographs presented in Figure 2, it can be observed that the foam samples formed cellulose materials and presented an open pore structure in which the pores were irregular and interconnected. The internal structure of all samples accorded according with their fibrous composition and bulk density.

The relative prediction error diagrams in Figure 6 and Figure 7 highlighted both the microstructural model, D-B, and the phenomenological model, JCAPL. Both models provided a good approximation of the experimental results for a wide range of frequencies. Comparatively, the JCA model presented better results for the frequencies within the midrange of 3–7 kHz. Considering that JCAPL is a corrected JCA model for low frequencies, the gained approximations of simulation to impedance tube tests can be accepted as a proper starting point for extended computational investigations. It is obviously difficult to underline a single theoretical model, which would be able to provide better results within the entire range of incident noise frequency. However, keeping all three models together, they supplied a good way to characterize the global soundproofing performance of FCM materials. 

The large variations in the relative prediction error (in a range of 0–0.2), even for the frequencies framed by the availability of each empirical model, can be explained by the aspects as follows. The constants used for the D-B model-based simulation have not been modified from the basic values. This assumption was adopted in order to evaluate the capability of any available extensive models that could possibly simulate FCM poroacoustics behavior. Therefore, an aim of this analysis was accomplished, and, in addition, was identified as a possible future direction of research related to a multi-criteria correlative analysis between the experiment and the D-B model-based estimation, which could optimize the values of *C*_1..9_ coefficients according to this type of materials.

The second aspect is that the simulations with phenomenological models (i.e., JCA, JCAPL) were based on a set of average-valued parameters. Further, experimental test results and the literature provided empirical expressions. This assumption was justified by the difficulties in the experimental evaluation of some parameters within phenomenological models, some of which still lack experimental methods, but benefit from the literature in terms of their theoretical approach. Using this assumption, the authors were able to implement these advanced poroacoustics models into the simulation procedure and therefore accomplished another aim of the research. Obviously, we identified another potential future direction of the investigation related to both the identification of a new feasible experimental method, as wall as the harmonization of the empirical formulations to the experimental datum. This improved the correlation of theoretical approaches with real evaluations.

Other than the previous discussions, the comparative analysis between experimental and computational results, especially for reflection coefficient estimated by the D-B and JCAPL models (see diagrams in Figure 7), have shown a relative error under 0.15 for the entire frequency range, with two singular local exceptions (E2 vs. D-B around 5 kHz and E1 vs. JCAPL around 9 kHz). 

Some local differences, comparatively provided by the empirical models to the experiments, are justified by the initial differences between the two experimental datum sets. Taking into account that each impedance tube supplies a specific frequency range of confidence, we have to conclude that the results provide relevant information about the noise insulation characteristics of these FCM materials.

Considering the extended computational investigations, comparatively presented in Figure 8, it can be observed that grouped evolutions for low to midrange frequencies had a differential behavior toward high frequency range. This evolution is more evident for the JCA model-based estimations. Nevertheless, parametric estimations in respect to the incidence angle have shown evolutions irrespective of the model. An interesting aspect derived from Figure 8 is that both absorption and the reflection coefficient denotes an extension of the sound insulation characteristic, leading towards low frequencies, in respect to increasing of the incidence angle (relative to normal direction of FCM layer). This tendency is available for low and midrange frequencies, up to 6–7 kHz. Beyond this limit, we observed a change of the tendency to increase incidence, around the 45º angle. Therefore, for frequencies greater than 7 kHz, the absorption performance grows up with the incidences of up to 45º and decreases for incidences above this value, even if the absorption absolute value becomes higher than the correspondent of normal incidence does.

It has to be mentioned that the simulations developed within this study created a perfect homogenous layer of FCM materials. This approach was based on the main hypothesis that, for accurate computational estimations, it had to acquire proper valued parameters and the simulation models had to be harmonized with available realistic behavior of such materials. This assumption and, obviously, the results within this study, are very important, because it provide the necessary basis for other computational approaches regarding different configurations of FCM layers within acoustic barriers. In practice, it is usually difficult to ensemble FCM-based soundproofing devices that maintain constant geometrical parameters of the layer. The structure of sound absorbent layer becomes usually affected by air inclusions with various shapes and dimensions. Thus, another potential future research direction is to investigate noise insulation characteristics of foam-formed cellulose materials that consider random structural air inclusions into the basic layer.

## 5. Conclusions

The cellulose loose-fill composite materials obtained by the aforementioned foam-forming methods represent an alternative solution for soundproof, especially compared with applications obtained using conventional methods. Furthermore, this method can be applied on both virgin and recycled cellulose fibers.

Considering that each impedance tube supplies a specific frequency range of confidence, we conclude that the presented results, on the whole, provide relevant information about the sound insulation characteristics of foam-formed cellulose materials.

The results presented in this paper are in good agreement with computational and experimental results, and provide extended soundproof characteristics to the incidence angle of the acoustic field. Further, our results supply additional information useful for future analyses of how air inclusions influence the FCM layer.

Herein, our findings provide a theoretical basis for computational investigations and a good starting point for future developments into the area of cellulose-based materials proper for soundproofing applications. These conclusions are justified by both the innovative foam-forming method and the valorization of recycled cellulose fibers. 

## Figures and Tables

**Table 1:** Fibrous components of foam formed cellulose materials. FCM: foam-formed cellulose materials; BHCF: bleached hardwood cellulose; RCF: recycled fibers from recovered papers.

FCM Sample Codes	Composition
P1	100% (BHCF)
P3S	100% (RCF)
P4	50% (BHCF) + 50% (RCF)

**Table 2:** Empirical constants for Delany–Bazley (D-B) model [30].

Material Type	C1	C2	C3	C4	C5	C6	C7	C8
material 1	0.0571	0.745	0.087	0.732	0.0978	0.700	0.189	0.595
material 2	0.078	0.623	0.074	0.660	0.159	0.571	0.121	0.530
material 3	0.114	0.369	0.0985	0.758	0.168	0.715	0.136	0.491
material 4	0.212	0.455	0.105	0.607	0.163	0.592	0.188	0.544

material 1: Rockwool/fiberglass; material 2: Polyester; material 3: Polyurethane foam of low flow resistivity; material 4: Porous plastic foams of medium flow resistivity.

**Table 3:** Tortuosity values for the FCM samples.

Tortuosity	Sample P1	Sample P3S	Sample P4
theoretical	1.008097	1.007585	1.00861
experimental (averaged values)	1.04721428	1.036407	1.049156122

**Table 4:** Airflow resistivity values for the FCM samples.

Airflow Resistivity [Pa s m^−2^]		Sample P1	Sample P3S	Sample P4
Theoretical estimation—Equation (29)		5936.869	5685.805	6161.118
Theoretical estimation—Equation (30)		5179.872	4538.770	5865.454
Experimental investigation (averaged values)		6104.445	5849.473	6332.070
Average value per sample		5740.395	5358.016	6119.547
Relative error [%] of experimental value related to	Equation (29)	2.75	2.80	2.70
	Equation (30)	15.15	22.41	7.37
Relative error [%] of average value related to	Experiment	5.96	8.40	3.36
	Equation (29)	3.31	5.77	0.67
	Equation (30)	10.82	18.05	4.33

**Table 5:** Characteristic parameters used within the computational investigations of FCM.

Parameter	Sample P1	Sample P3S	Sample P4
Bulk density [kg m^−3^]	37.3	36.3	38.18
Foam porosity [-]	0.984	0.985	0.983
Flow resistivity [Pa s m^−2^]	6000	5770	6200
Tortuosity [-]	1.0081	1.0076	1.00861
Static viscous tortuosity [-]	1.3441	1.3435	1.3448
Static thermal tortuosity [-]	1.3333	1.3333	1.3333
Viscous characteristic length [m]	157.24 × 10^−6^	160.22 × 10^−6^	154.80 × 10^−6^
Thermal characteristic length [m]	314.48 × 10^−6^	320.44 × 10^−6^	309.60 × 10^−6^
Static thermal permeability [m^2^]	3.02 × 10^−9^	3.14 × 10^−9^	2.92 × 10^−9^
